# MECHANISMS IN ENDOCRINOLOGY: Anorexia nervosa and endocrinology: a clinical update

**DOI:** 10.1530/EJE-18-0596

**Published:** 2018-10-25

**Authors:** René Klinkby Støving

**Affiliations:** 1 Nutrition Clinic, Center for Eating Disorders, Odense University Hospital; 2 Endocrine Elite Research Centre, Institute of Clinical Research, University of South Denmark, Faculty of Health Sciences; 3 Psychiatric Services in the Region of Southern Denmark, Odense, Denmark

## Abstract

Anorexia nervosa is a syndrome, that is collections of symptoms, which is not defined by its etiology. The severe cases are intractable. The syndrome is associated with multiple, profound endocrine alterations which may be adaptive, reactive or etiologic. Adaptive changes potentially may be inappropriate in clinical settings such as inpatient intensive re-nutrition or in a setting with somatic comorbidity. Electrolyte levels must be closely monitored during the refeeding process, and the need for weight gain must be balanced against potentially fatal refeeding complications. An important focus of clinical research should be to identify biomarkers associated with different stages of weight loss and re-nutrition combined with psychometric data. Besides well-established peripheral endocrine actions, several hormones also are released directly to different brain areas, where they may exert behavioral and psychogenic actions that could offer therapeutic targets. We need reliable biomarkers for predicting outcome and to ensure safe re-nutrition, however, first of all we need them to explore the metabolism in anorexia nervosa to open new avenues with therapeutic targets. A breakthrough in our understanding and treatment of this whimsical disease remains. Considering this, the aim of the present review is to provide an updated overview of the many endocrine changes in a clinical perspective.

## Invited Author’s profile


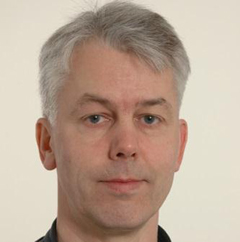


Rene Klinkby Støving, MD, PhD, is an endocrinologist and professor in eating disorders, University of Southern Denmark, and Mental Health Services in the Region of Southern Denmark. He hasextensive clinical experience in anorexia nervosa having contributed to the establishment of an interdisciplinary center at Odense University Hospital in 1993. In 2010, he established the only specialized endocrine unit in Denmark for patients with severe anorexia nervosa. His research areas are clinical endocrinology and epidemiological studies of anorexia nervosa.

## Introduction

Anorexia nervosa (AN) is a syndrome as it describes a collection of symptoms and is not defined by an etiology. It is characterized by disturbed body image, ego-syntonic neglect, ambivalence, self-starvation, loss of body weight, obsessive thoughts of food, ritualistic patterns of food intake, elevated physical activity, depression, anxiety and emotional rigidity. The severity varies from mild subclinical cases to chronic enduring and fatal cases. The diagnostic criteria were revised in 2013 for the Fifth Edition of the Diagnostic and Statistical Manual (1), leading to markedly increased diagnostic heterogeneity and a 30–50% increase in lifetime prevalence (2, 3), which is estimated to be 1–4% (4). Diagnostic boundaries, which are based on symptoms, may not reflect the underlying pathophysiological mechanisms. The evidence base for treatment is still very limited (5). There was no evidence that the prognosis has improved throughout the 20th century (6). However, since that the outcome may have improved with an evolutionary neuroscience approach to treatment (7).

AN is associated with multiple, profound endocrine disturbances (8, 9, 10), and adaptation to chronic semi-starvation allows survival even with extreme emaciation (11). The endocrine alterations can be adaptive, reactive or etiologic. For instance, hypothalamic amenorrhea is often secondary, but it can precede the weight loss and persist for a long time after weight and motor activity have returned to normal (12). Hypersecretion of corticotropin-releasing hormone (CRH) is a response to starvation. But at the same time, it may maintain and intensify the anorexia, physical hyperactivity and amenorrhea, thus potentially creating a vicious circle, although the latter remains to be proven. Twin studies uniquely have supported a genetic origin for the observed familial accretion in AN. Hypothetically, the same genetic factors that influence normal variation in BMI may also influence dysregulation of appetite and weight-related features in AN. This hypothesis was supported by a recent genome-wide association study which reveled genetic correlations with metabolic traits in terms of regulation of insulin, glucose and lipid (13).

The purpose of the review is to provide an updated overview of the many endocrine changes with focus on the clinical consequences. For more in-depth discussion the article refers to a number of literature reviews each of which deals with specific hormone systems in AN. It is a narrative review that is combined with my own experiences from over 25 years of clinical work with AN. The review does not consider other eating disorders than AN, and it predominantly focuses on severe illness. As several comprehensive reviews on bone metabolism in AN have been published in recent years (14, 15, 16, 17, 18), this aspect is omitted from the present review.

## The refeeding syndrome

### Adaptation

Natural selection has led to the evolution of hormonal systems that facilitate appropriate short-term and long-term equilibrium between energy expenditure and food intake during episodes of starvation.

Chronic and severe AN is endocrinologically characterized by an extreme adaptive mechanism that allows the patients to survive. The overall endocrinology of chronic starvation is well described (19) and is characterized by increased secretion of cortisol and growth hormone (GH) and suppressed levels of leptin, sex hormones and T3. In AN, a BMI as low as 7.8, corresponding to 65% below expected body weight, has been survived without severe sequelae (11). Since the publication of that case report, the same patient relapsed with a weight drop to BMI 7.2, which she also survived (unpublished data). This degree of emaciation far exceeds what has ever been reported in hunger strikes and hunger disasters. However, the adaptation represents a considerable risk during early re-nutrition, termed the refeeding syndrome (RFS). Refeeding in AN with serious or fatal complications is anecdotally recognized (20, 21, 22, 23, 24, 25). RFS is defined as electrolyte disturbances, principally low serum concentrations of intracellular ions such as phosphate, magnesium and potassium (26, 27). The electrolyte movements from the extracellular to the intracellular space is believed to be caused by insulin spikes (28). Thiamine (vitamin B1) deficiency is also associated with RFS (29) and manifests as Wernicke’s encephalopathy syndrome and lactic acidosis. The clinical manifestations of RFS and hypophosphatemia are cardiac failure or arrest, hemolytic anemia, delirium, seizures, coma and sudden death (27, 30). To avoid RFS, a very low initial energy level is prescribed. An unintended consequence of this approach, however, is additional weight loss or the underfeeding syndrome (http://www.rcpsych.ac.uk/files/pdfversion/CR162.pdf.). It is currently debated whether further initial restriction in carbohydrates (to prevent insulin peaks) may allow a higher energy intake early on (31). A systematic review illustrated that 26 out of 27 refeeding studies were observational and limited by bias by indication, leading the authors to conclude that there is presently no evidence to require changes to the current guidelines (32).

### Clinical consequences

In severe AN, electrolyte levels must be closely monitored during the refeeding process, and the need for weight gain must be balanced against the potentially fatal complications, i.e. ‘Start low and advance slow’ (http://www.rcpsych.ac.uk/files/pdfversion/CR162.pdf, MARSIPAN. Accessed April 15, 2015). However, an overestimation of the risk may have significant clinical negative consequences. Maybe the definition of a ‘high risk’ patient is too conservative. If there is no active purging or somatic comorbidity, the arbitrary definition for high risk in adults maybe should be adjusted to below BMI 12–13. Controlled randomized studies are required.

## Glucose metabolism

### Adaptation

Insulin-induced hypoglycemia was used therapeutically in AN until the 1960s and represents one of the bizarre inventions in medical history (33, 34). In general, fasting levels of glucose are generally lower in AN than in controls (35, 36). Sudden unexpected death is still well described among young women with AN, and manifest hypoglycemia was recognized as the cause of death in some of these fatal cases (37). Hypoglycemic coma in AN can be refractory or repetitive, so that continuous glucose administration is required to maintain euglycemia (38, 39, 40, 41, 42). The incidence of asymptomatic or subclinical hypoglycemia is unknown. Hypoglycemia may be related to liver injury, which is well described in AN, due to protein catabolism, dehydration, anemia, hypotension, ischemia and autophagy (43, 44). Marked glycogen depletion is a frequent finding in liver biopsies (45), and the severity of liver damage predicts low glycogen storage and thus increased risk of hypoglycemia (46). Furthermore, liver enzymes may rise during the early phase of re-nutrition (47). Psychopharmacologic treatment with olanzapine has been associated with fatal hypoglycemia in AN (48).

The secretion of GH (see below) and cortisol (see below) are elevated in AN, and both are known to induce insulin resistance and thus counteract the tendency for hypoglycemia. Studies of insulin sensitivity in AN have provided contradictory results, from unchanged (49) to increased (50, 51, 52) or decreased (53). These conflicting results are related to the different techniques used to evaluate insulin sensitivity and to different disease stages not only in terms of actual BMI, but also the short-term nutritional status. Using a hyperinsulinemic–euglycemic clamp, it was observed that refeeding was associated with onset of insulin resistance (54).

Temporary stress diabetes has only been reported as individual cases (55). It is observed occasionally in my specialized unit, but to my knowledge there are no published reports of how often and under what circumstances stress diabetes occurs in hospitalized patients with AN. Gastrointestinal hormones such as glucagon-like peptide-1 (GLP-1) and amylin are released in response to a meal and probably limit the size of an ongoing meal. GLP-1 is secreted from the L cells in the mucosa of the distal intestinal tract and stimulates secretion of insulin. Amylin is co-secreted with insulin from pancreatic beta cells. The plasma levels of GLP-1 (56, 57, 58) and amylin (35, 53, 59) are both found to be low in AN, which seems to be appropriate in relation to adaptation.

### Clinical consequences

The frequency of hypoglycemia and its relation to weight loss, refeeding syndrome, diagnostic subtype or other clinical characteristics has not been fully studied. Blood glucose should be monitored regularly in severe AN, especially during the night or in relation to exercise. In some centers, hypoglycemia is prevented by supplementary intravenous 10% glucose (20–40 mL/h) in the first days of refeeding (60); however, as discussed earlier, this may increase the risk of refeeding syndrome. Therefore, prophylactic 5% dextrose is used in other centers (61).

## Hypothalamic–pituitary–adrenal axis (HPA axis)

### Adaptation

Adrenocorticotropic hormone (ACTH) is increased together with high levels of cortisol, indicating a state of CRH hypersecretion. This is further supported by normal cortisol response to stimulation with ACTH (62, 63) but a weakened response to stimulation with CRH (64, 65). Finally, CRH hypersecretion is confirmed by measurements in cerebrospinal fluid from patients with AN (64, 66). This has been evident since the 1980s. As ACTH and opioids are derived from the same prohormone (proopiomelanocortin, POMC), ACTH secretion is preceded by activation of the POMC system. The opioid system may influence hedonic eating behavior (67) and may also stimulate the urge to exercise (68). Both physical stress and psychological stress activate the HPA axis with secondary anorectic effect. In healthy individuals, a high glucocorticoid level may induce anxiety as an appropriate coping response in acute stressful events. In AN, CRH hypersecretion may be a compensatory mechanism for a state of cortisol resistance. Many animal studies show that central microinjections of CRH can lead to anorexia and increased motor activity (69) that can be reversed by CRH antagonists (70). Somatostatin counteracts the anorectic action of CRH on food intake (71), and there is evidence that the somatostatinergic tonus is impaired in AN (72), suggesting that low hypothalamic somatostatinergic activity could also be pathogenic. In weight-recovered patients, the dexamethasone suppression test predicted a continuing stress condition and high risk of relapse (73).

The hippocampus belongs to the limbic system and is a critical structure for memory functions. It contains many glucocorticoid receptors and thus may be vulnerable to long-term stress. In fact, neuroimaging studies have clearly shown that hippocampal atrophy is related to chronic stress and hypercortisolemia in studies on Cushing's syndrome (74), depression (75, 76) and AN (77).

Neurosteroids are steroids that are synthesized de novo in the brain. They are supposed to exert a negative feedback on the HPA axis and to be implicated in anxiety disorders (78). Neurosteroids may have important therapeutic potential (78), but this remains to be clarified. So far, studies on neurosteroids in AN have been conducted on circulatory levels only (79) and cannot reveal true cerebral effects.

Cortisol stimulates gluconeogenesis, and cortisol levels in AN are shown to be inversely correlated to fasting glucose (80). This suggests that increased cortisol secretion (like increased GH secretion) in AN is an adaptive mechanism to maintain euglycemia (see section above).

### Clinical consequences

CRH may be involved in a potential vicious circle in AN (8). However, so far, the cause–effect relationship remains unclear and needs to be resolved by further experimental analysis. Thus, the therapeutic potential of cortisol suppressants should be further explored. This was explicitly discussed in a recent review (81). There is preliminary evidence that anti-glucocorticoid therapies could be useful in the treatment of major depression (82, 83, 84), and the degree of cortisol elevation was related to depression and anxiety measures in 18 patients with AN (85). Mifepristone is a potent progesterone primarily used for medical abortion (86). In addition, it is a competitive glucocorticoid antagonist that is used therapeutically in Cushing’s syndrome (87). The effect of mifepristone in AN has only been explored in a short-term study of eight patients, which found that mifepristone increased urinary free cortisol excretion and early morning cortisol plasma levels (88). To my knowledge, the effects of cortisol synthesis inhibitors, like ketoconazole and metyrapone, have not been explored in AN.

## Hypothalamic–pituitary–thyroid axis

### Adaptation

Low levels of thyroid hormone decrease resting energy expenditure (REE) (89). Self-starvation and weight loss is followed by adaptive decline in circulating concentrations of T3, T4 and thyroid-binding globulin (TBG) to downgrade the metabolic rate and REE (90, 91) (Low T3 Syndrome). Reverse T3 is elevated from increased peripheral deiodination of T4 (92, 93, 94). The TSH level is normal or slightly decreased, while the TSH response to exogenous TRH stimulation is blunted and delayed (95). During weight recovery, total T3 rises with the rising metabolic rate (96). In AN the thyroid gland is atrophic even at normal TSH levels (97).

### Clinical consequences

It may be useful to monitor T3 levels during a treatment course to assess metabolic adaptation and recovery goal weights.

A relationship between AN and depressive symptoms is well established. Moreover, there is higher prevalence of primary affective disorders in the relatives of AN patients, and evidence for common genetic factors has been provided (98). Thyroid disorders are associated with affective disturbances, which may persist even after appropriate substitution (99). Exogenous administration of thyroxine, TSH or TRH was found to exert an antidepressant effect or to amplify the antidepressant effect of imipramine in primary affective disorders (100, 101). However, evidence is lacking for causal relationships and yet there is no evidence to support a beneficial therapeutic effect of thyroid hormones in AN. On the contrary, it would probably increase the cardiovascular risk by eliminating the adaptive bradycardia and low REE. Finally, there is a problematic abuse potential of thyroxine treatment in AN. This may be overlooked, as it is not addressed in the literature except for a single case report (102).

## Hypothalamic–pituitary–gonadal axis

### Adaptation

From an evolutionary perspective, it is fitting that the reproductive system shuts down in times of hunger and stress. In adult women with AN, earlier studies have shown immature, prepubertal, low-amplitude LH pulses that reverse with weight gain (103). The altered LH pulsatility manifests as hypothalamic amenorrhea. The immature LH secretion pattern can also be induced in experimental starvation of healthy subjects (104). The gonadotropin secretion is stimulated by estradiol (105) and leptin (106), which are both decreased in AN. In addition, increased ghrelin and cortisol levels both suppress gonadotropin secretion during starvation in ED (107, 108).

The pronounced skewed gender distribution in AN gives rise to considerations of sex-dependent susceptibility and potential pathogenic roles of the sex hormones. Estradiol modulates central processes of both satiating and orexigenic peptides (109). Neuroendocrine sex differences in regulation of eating behavior have been thoroughly reviewed (110, 111). Recently discovered hypothalamic peptides kisspeptin, gonadatropin-inhibitory peptide and amide-related peptide-3 all seem to be involved in the genesis of stress-induced hypothalamic amenorrhea (112), but their potential role in AN remains uncertain.

### Clinical consequences

In the DSM-5 criteria, amenorrhea is no longer required for the diagnosis of AN (1) although menstrual resumption remains a clinical hallmark of recovery (https://www-nice-org-uk.proxy1-bib.sdu.dk/guidance/ng69. Eating disorders: recognition and treatment. National Health Institute for Care Excellence (NICE) 2017). Secondary amenorrhea may have many causes, including underweight. Menstrual resumption typically fails in weight recovered AN patients due to anxiety or excessive exercise. For example, in a study of 100 adolescent girls with AN, amenorrhea persisted in 15% of the women despite recovery of normal body weight (113). In a 1-year follow-up study of 57 patients who had reached a BMI of at least 18.5, only 35% had resumed menstruation (114). This is in line with a study of 113 adult women who did not use oral contraceptives and had a history of AN (12). The predicted probability of resuming menses at various BMI levels and body fat percentages was calculated, and at a BMI of 19 or a body fat percentage of 23, only 50% of the subjects were expected to resume their menstrual function (12). Furthermore, body composition measured by dual energy X-ray absorptiometry was not superior to BMI in predicting menstrual recovery in AN (12). In a small minority of patients, menstruation may recover at a quite low BMI; in a few cases, even lower than BMI 14 (12).

In athletic women with slight underweight and hypothalamic amenorrhea, administration of exogenous leptin increased pulsatile LH, resulting in an enlargement of the ovaries, increased number and sizes of dominant follicles and elevated plasma estradiol levels (115). Three of eight subjects achieved ovulatory menstrual cycles with this leptin therapy (115). In AN, however, increased leptin did not uniquely predict menstrual resumption (116), and as exogenous leptin also reduces fat mass and appetite, this is not an option in treatment of AN.

Both estrogen and testosterone play important and well-known roles in maintaining muscle, mucous membranes and bone mass. Some of these consequences of starvation-related atrophy could theoretically be alleviated by substitution with estradiol. However, several systematic reviews examining the effect of oral estrogen preparations on bone health in women with AN consistently found insufficient evidence to support this (14, 117). The lack of favorable effects could be associated with estrogen’s suppressive effect on hepatic synthesis of IGF-1, although transdermal estrogen did not suppress IGF-1 (118). In line with this, an 18-month RCT showed that transdermal physiological estrogen replacement with cyclic progesterone increased bone accrual rates in adolescents with AN (119).

Anxiety and depression are well-known features of AN, and it is possible that deficiency in sex hormones worsens these symptoms in AN. For instance, hypoandrogenemia has been associated with more severe depression and anxiety in women with AN independent of body weight (120). In adolescent girls with AN, transdermal estradiol improved the tendency to experience anxiety independently of weight changes, but it did not affect attitudes toward eating, eating behavior or body shape perception (121).

Unplanned pregnancies appear to be relatively common in AN (122, 123). Reports of increased risk for miscarriages, cesarean deliveries, premature births and perinatal lethality in women with a history of AN indicate the need for careful monitoring of these women during pregnancy and of their children (114, 124).

## Growth hormone (GH) axis

### Adaptation

The metabolic effects of GH are related to its pulsatile secretory pattern, and AN is a state of acquired GH resistance. Secretory burst frequency, burst mass and burst duration are increased in AN, with markedly higher daily pulsatile GH secretion and basal (non-pulsatile) GH secretion (125). Furthermore, women with AN had significantly higher GH approximate entropy scores than controls, denoting marked irregularity of the GH release process (125). The alterations in GH secretion appear to be correlated with the weight loss in AN and are reversed by refeeding (125, 126). GH pulsatility is affected by sleep and vice versa (127). Insomnia is a recognized symptom in AN and should also be considered in the treatment. Circulating levels of total ultra-filtrated (128) and bioactive (50) IGF-1 levels are greatly decreased in AN. As GH is a potent stimulator of gluconeogenesis, a beneficial effect of the higher GH and lower IGF-1 levels is maintenance of euglycemia (see above). The state of GH resistance in AN is thus adaptive through its gluconeogenic role (through increased lipolysis) to maintain euglycemia in a state of low energy availability (129). Fasting stimulates the secretion of fibroblast growth factor 21 (FGF21) from hepatocytes, adipocytes and myocytes, and FGF21 inhibits transcription 5 which is a mediator of intracellular GH effects (130). Expectedly in AN, the level of FGF21 is increased and is inversely correlated with the level of IGF-1 (131).

Placental-associated protein protein-A (PAPP-A) is abundant in the circulation of pregnant women and is widely expressed in multiple tissues. By cleaving a subset of IGF-binding proteins, PAPP-A functions as a growth-promoting enzyme within the tissues near the IGF receptor and releases bioactive IGF (132). To my knowledge, however, PAPP-A has not yet been studied in AN.

### Clinical consequences

The therapeutic effects of GH in AN has only been scantily investigated. Ten girls with early-onset AN and subsequent growth failure and delayed puberty were treated with rhGH for 2 years (133). Increase in height was observed, but as there was no control group, it is not possible to separate the potential effect of rhGH from that of the nutritional interventions (133). Intranasal GH-releasing peptide-2 was administrated to one patient with severe and enduring AN who then increased body weight from 21 to 28 kg over 14 months (134). In an RCT ten patients with BMI 17.4 ± 0.4 self-administered GH for 12 weeks (135); however, supraphysiologic doses greater than five times the dose used to treat GH-deficient patients did not increase the level of IFG-1 (135). Importantly, the treatment led to a further decrease in fat mass, which can hardly be of therapeutic benefit in cachexia. Furthermore, the safety of GH treatment in hypercatabolic GH-resistant patients has been severely questioned by a multicenter trial in critically ill patients in intensive care units (136).

## Ghrelin

### Adaptation

Approximately 70% of circulating ghrelin is secreted by gastric cells. Ghrelin cross the blood–brain barrier and exerts multiple physiological effects that go far beyond its initial characterization as a GH secretagogue. Its strongest effect is stimulation of food intake via activation of orexigenic pathways produced by the two neuropeptides, agouti-related protein (AgRP) and neuropeptide Y (NPY). These pathways are essential for normal feeding behavior, as evidenced by the rapid arrest of feeding when these neurons are ablated in mice (137). Circulating ghrelin is present in acylated and desacylated forms, and only acylated ghrelin stimulates appetite (138). Oacyltransferase (GOAT) is the only known enzyme capable of acylating ghrelin *in vivo*, as indicated by the absence of acyl ghrelin in mice lacking GOAT (139). Desacyl ghrelin may act as a separate hormone through an unknown receptor (140). The broad pharmacological potential of ghrelin pathways was recently prominently reviewed (141, 142, 143).

Numerous studies have shown that the circulating level of ghrelin is elevated in AN and the plasma concentration decreases with weight gain (50, 144, 145, 146). Moreover, the secretory pulse amplitude is increased (147, 148). Obestatin is cleaved from acylated ghrelin, and the plasma level of obestatin is also increased in AN (149). Some studies have reported differences in ghrelin levels in restrictive and binge-purge subtypes of AN (150), and short-term weight gain led to an increased ratio of acylated/desacylated ghrelin. So far, genetic association studies have failed to reveal any polymorphism in ghrelin secretion as a potential etiologic factor in AN (151, 152).

To my knowledge, only one experimental intervention study has been published. In a pilot study of five patients with restrictive severe AN (BMI 10.2–14.6), supraphysiologic doses of ghrelin (3 μg/kg) were infused twice a day preprandially for 2 weeks (153). During this intervention, hunger measured by a visual analog scale increased in four of the five patients, whereas body weight decreased in three of the five patients (153). Appetite measurement by visual analog scale has not been validated in AN, and due to lack of a randomized, placebo-controlled group and blinding of the investigators, conclusions from that study cannot be drawn but should inspire subsequent studies. In another study of 30 patients with severe AN, medium-chain triglyceride (MCT) supplementation increased the level of acylated (active) ghrelin levels in a dose-dependent manner (154). However, the increase in body weight did not differ significantly between low, moderate and high MCT supplementation (154).

### Clinical consequences

Increased ghrelin secretion seems an appropriate adaptive appetite-stimulating response to chronic starvation.

In theory, ghrelin agonism offers an approach in treatment of loss of appetite. Small RCTs in cachexia related to cancer and chronic obstructive pulmonary disease show promising results (155, 156, 157). Gastroparesis, nausea and other forms of gastrointestinal discomfort is very common in AN (158). An RCT with the ghrelin receptor agonist, ulimorelin, demonstrated symptomatic improvements over placebo in 23 hospitalized patients with diabetic gastroparesis (159). The ghrelin agonist, relamorelin, was recently tested in an RCT of 22 chronic, stable patients with AN (BMI 17.7 ± 0:4) (160), where 3 out of 10 patients dropped out due to an increased hunger sensation. Gastric emptying was shortened by the ghrelin agonist; however, there was no significant weight gain during 4 weeks of treatment (160). Despite this, there is obviously basis for further exploration of the therapeutic potential of ghrelin agonism in AN.

## The endocannabinoid system

### Adaptation

A search for ‘medical cannabis’ gives today more than 6 million hits in Google and demonstrates a huge public interest and debate. Old studies of tetrahydrocannabinol (THC) intake in humans described short-term effects in terms of elevated hunger ratings and increased food intake (161, 162). However, in cancer (163) and AIDS-related (164) cachexia, clinical trials have shown only minor weight gains.

The endogenous cannabinoid system has been characterized in detail. The system has a role in feeding-related neural and hormonal circuitry at several levels, both central and peripheral, being related to integrative functions (hypothalamus and hindbrain), hedonic evaluation of foods (limbic system), gut signaling (intestinal system and pancreas) and adipogenesis (liver and fat tissue). It was recently shown that in eating disorders, the availability of the cannabinol receptor 1 was negatively correlated with BMI throughout the mesolimbic reward system (midbrain, striatum, insula, amygdala and orbitofrontal cortex), which constitutes a pathway implicated in processing appetitive motivation and hedonic food rewards (165). The hedonic roles are of special interest in AN (166) in view of the consensus that patients with AN suffer from universal anhedonia (167) and interoceptive impairment.

Patients with AN have high peripheral concentrations of the endocannabinoids anandamide, 2-arachidonoylglycerol, oleoylethanolamide and palmitoylethanolamide (166, 168). Moreover, seven weight-recovered AN patients differed from healthy controls in peripheral endocannabinoid response to meals (166). So far, however, endocannabinoids have only been measured in a few small populations by a few research units. An increased level could be interpreted both as an appropriate (but insufficient) adaptation to malnutrition or as dysregulated reward processes.

### Clinical consequences

The therapeutic perspectives in AN have previously been reviewed (169). In an old study, the effects of diazepam and high doses of THC were compared in a non-blinded randomized trial of 11 patients with AN (170). The weight gain in the THC group was slightly higher than that in the diazepam group, although the participants were occasionally tube-fed during the trial (170). An add-on, randomized double-blind, controlled crossover study of 24 patients with severe and enduring AN found that low-dosage dronabinol (10 mg daily) over 4 weeks had a statistically, but hardly clinically, significant weight-gaining effect (171). It should be noted, however, that the highest weight gain was observed in the last week of that trial (171), indicating that treatment should have been extended beyond the 4 weeks. During the 4-week treatment, dronabinol was associated with increased physical activity monitored by accelerometer (172). This contrasts with animal experimental studies (173) and a recent clinical case study using a higher dose of dronabinol (daily 15 mg), which alleviated the urge to be physically active (174).

The two small, preliminary clinical trials conducted so far have not confirmed a therapeutic potential for cannabinol in AN. However, they have not ruled out a beneficial effect either. Further explorative research should thus be done, especially in patients with severe and enduring AN. There may still be prospects for cannabinol in modulation of both feeding behavior and the anxiety, anhedonia and motor restlessness in AN.

## Vasopressin

### Adaptation

Vasopressin is also referred to as antidiuretic hormone, arginine vasopressin and argipressin. Hyponatremia is very common in AN (175) and may lead to complications such as vomiting, altered level of consciousness and seizures. Purging behavior is the dominant cause (176, 177); although the syndrome of inappropriate antidiuretic hormone secretion (SIADH) may also be implicated (178, 179, 180, 181). A study of 12 patients (BMI 15.2 ± 0.6) showed altered osmoregulation both at baseline (normal ADH levels despite lower plasma sodium and osmolality) and after water deprivation (lower urinary concentrating ability) (182). The study population was heterogenic in terms of pharmacologic treatment with antidepressant and oral contraceptives, however, which confounds pathogenic interpretation (182). Partial diabetes insipidus has also been documented in AN due to defective vasopressin secretion, with clinical symptoms of polyuria and polydipsia (181).

In addition to the established endocrine effects on osmoregulation and arteriole constriction, vasopressin is released directly into the brain where it is considered to be involved in social behavior, sexual motivation, depression and responses to stress (183). Animal experimental evidence of this is thoroughly reviewed (184). In humans, depression has been found to be associated with elevated levels of ADH in cerebrospinal fluid and enhanced pituitary sensitivity to ADH (185). However, in a prospective study of soldiers (*n* = 907) deployed to combat zones, the plasma level of vasopressin was not related to the development of post-traumatic stress syndrome over time (186). The potential pathogenic role of vasopressin in anxiety and depression in AN remains to be examined and has had little attention in AN research.

### Clinical consequences

The cause of every case of hyponatremia must be carefully resolved, as correctional therapy will differ. It is particularly important to recognize hypotonic dehydration since fluid restriction in this situation will have serious consequences (187). Antidepressants are often prescribed in AN although the evidence is lacking (188), and SIADH resulting in hyponatremia is a frequent adverse effect.

It has been suggested that ADH and oxytocin (see section below) levels could be biomarkers for psychiatric disease; however, this could not be confirmed in a recent meta-analysis (189).

## Oxytocin

### Adaptation

Besides the established peripheral endocrine actions of oxytocin secreted from the posterior pituitary gland, it is well documented that oxytocin is also released from centrally projecting neurons directly to brain areas. In numerous human experimental studies, intranasal application of oxytocin has been claimed to exert psychological effects such as attachment (190), generosity and empathy (191, 192). Furthermore, oxytocin may possess anxiolytic and antidepressant effects (192) and affect eating behavior as well (193). Several thought-provoking trials were recently critically reviewed for publication bias and questionable *post hoc* interpretations (194). It was also questioned whether peripheral measurements of oxytocin reflect central release (194).

In patients with binge eating disorder and obesity (*n* = 7), an 8-week RCT with nasal oxytocin application combined with an energy-restricted diet showed no significant reduction in binge eating or body weight (195). Studies on oxytocin in AN have reported lower circulating levels (196, 197, 198) and lower concentrations in CSF that return to normal during refeeding (199). Increased postprandial serum level of oxytocin in 13 patients (BMI 17.7 ± 0.3) has been suggested to be adaptive to decrease the anxiety induced by meals (200). SNPs of the oxytocin receptor gene have been investigated in a few studies. One study reported higher methylation of the receptor gene in AN (201) and associations with the severity of eating disorder symptoms (202).

### Clinical consequences

The clinical consequence of the above findings on oxytocin pathways in AN remains unknown. Animal experimental data have not yet been translated to clinical human studies.

## Gut peptides

### Adaptation

Besides their many local effects on gastrointestinal motility and secretion, gut peptides act directly on neurons in hypothalamic and brainstem centers of appetite control and influence both short-term and long-term energy balance. The most well-studied are cholecystokinin (CCK), peptide YY (PYY), glucagon-like peptide-1 (GLP-1), oxyntomodulin and ghrelin; however, the list of identified gut hormones is very long and still grows. Except for ghrelin, all the mentioned hormones act to increase satiety and decrease food intake. For ghrelin, see the section above. In AN, the CCK level in the CSF remains unchanged (203) whereas there are contradictory reports about basal and postprandial serum concentrations of CCK (204, 205, 206, 207, 208). PYY is believed to play a role in meal termination and satiety, partly by reducing appetite stimulation by ghrelin (209). Plasma levels of PYY in AN are paradoxically reported to be increased in some studies (144, 210), but decreased in other studies (211, 212). GLP-1 is supposed to be a brain-gut peptide that acts as a hormone and neurotransmitter, mediating processes related to glucose metabolism and satiety. Like PYY, there are contradictory reports of the level of GLP-1 in AN (57, 58, 213). Oxyntomodulin is a product of the glucagon precursor that co-activates the GLP-1 receptor. To my knowledge, it has so far only been measured in one study of AN, although a control group was not included in that study (214).

The potential pathogenic roles of gut hormones in AN have been comprehensively reviewed, and it was concluded that most of the observed circulating alterations dissolved after restoration of body weight, indicating adaptive rather than pathogenic roles (215, 216). The many contradictory results could be due to variations in the short-term nutritive status, which is in fact not considered in any of the above-mentioned studies. Besides the humeral pathways, a potentially important player in the gut-brain axis is the gut microbiota. In recent years, several small studies have been published (217, 218, 219, 220) as well as 13 reviews focusing on the microbiota specifically related to AN (221, 222, 223, 224, 225, 226). The exploration of this essential area is still in its infancy. Caseinolytic protease b (ClpB) is an example of a peptide produced in the gut bacteria, and ClpB has been identified as a mimetic of α-melanocyte-stimulating hormone, a neuropeptide which are supposed to be involved in satiety and anxiety signaling (227). Remarkably, the plasma level of ClpB has been found to be increased in patients with AN (228).

### Clinical consequences

The gut peptides probably play a critical part in adaptation to starvation and refeeding, but their exact roles are not yet clarified. Speculatively, they might contribute to the maintenance of disordered eating behavior and may also influence the outcome.

## Adipokines

### Adaptation

Adipokines are cytokines that are secreted by adipose tissue and modulate energy expenditure, glucose homeostasis, and appetite regulation. Leptin is by far the most extensively studied. Reduced serum levels of leptin in AN (229) reflect decreased fat mass and appear to be an appropriate adaptation to reduce an anorexigenic stimulus that increases with refeeding and recovery (both free and bound leptin) (230, 231). Extensive and compulsive exercise is common in AN (232) and is not just compensatory to burn calories (233, 234). Starvation in animals has consistently been reported to induce motoric hyperactivity (235), and there is evidence that leptin deficit is implicated in this phenomenon as leptin administration prevents starvation-induced hyperactivity (236, 237). Although animal experimental data must be translated to humans with caution, from an evolutionary perspective, an increased drive for physical activity seems appropriate to facilitate finding food. This is supported by the finding that leptin levels were lower in men with exercise addiction compared to non-addictive exercisers, even when adjusted for body fat percentage (238). However, an accelerometric study of 61 hospitalized patients with AN (BMI 14.8 ± 2.0) did not reveal a clear association between leptin levels and physical activity (239).

The adaptive roles of adiponectin in AN have been thoroughly reviewed (240). Adiponectin exists in several isoforms with specific receptors (241) and has insulin-sensitizing properties. Serum levels are negatively correlated to BMI and body fat mass (242). In line with this, most studies found increased circulating levels of adiponectin in AN, which decrease during refeeding (52, 243, 244). In animal studies, intracerebroventricular administration of adiponectin has been shown to decrease body weight. In human studies, peripheral administration of adiponectin reduces body weight by enhancing energy expenditure and fatty acid oxidation and by reducing food intake (245). Based on these observations, it has been suggested that hyperadiponectinemia could contribute to the pathogenesis of AN (240, 246).

Visfatin is mainly synthesized in abdominal adipose tissue. Like the other adipokines, it is implicated in adipocyte differentiation and is metabolically active in energy and body-weight regulatory networks. In contrast to the circular levels of adiponectin and leptin in AN, the studies on visfatin in AN have so far produced conflicting results (247, 248, 249).

### Clinical consequences

The therapeutic potential of leptin is briefly discussed in the section above (section *Hypothalamic–pituitary–gonadal axis*). In addition, severe hypoglycemia can be a serious clinical challenge, as mentioned above (section *Glucose metabolism)*. Hypothetically, the increased level of insulin-sensitizing adiponectin may aggravate this problem (250).

## Myokines

### Adaptation

Myokines are produced and released by muscle cells in response to muscular contractions and, like the adipokines, they have autocrine, paracrine and endocrine effects (251, 252). The myokines are believed to represent a link between the myocyte and the beneficial mental effects of exercise. For instance, a meta-analysis of 42,264 persons indicated that exercise exerts antidepressant and anxiolytic effects (252).

Irisin, previously named fibronectin type III domain-containing protein 5, is a myokine involved in browning of white adipose tissue, making it metabolically active and capable of increasing thermogenesis. Irisin has been reported to be decreased in AN (253) and thus probably represents another adaptive mechanism to conserve energy through less browning of white fat. It is found not to be affected by exercise in patients with AN in contrast to healthy controls (254), possibly due to the muscle catabolism in these patients.

Brain-derived neurotrophic factor (BDNF) is one of the neurotrophic growth factors found in the brain, but it also belongs to the family of myokines. Intracerebral infusion of BDNF in animals enhanced physical activity and modulated thermogenesis (255). Studies on circulating levels of BDFN in AN have shown conflicting results (256, 257, 258), indicating that the serum levels are influenced by multiple factors such as nutritive status, exercise, and anxiety.

### Clinical consequences

At this point, myokines have no clinical consequences in AN. Several myokines remain to be studied in AN, e.g. myostain and follistatin, and they could prove to be important in our understanding of starvation-induced excessive exercise and anxiety in AN.

## Clinical Conclusions

Nearly all the endocrine systems are profoundly altered in severe AN. An important focus of clinical research in AN should be to identify biomarkers associated with different stages of both weight loss and refeeding, and then to assess their psychometric properties. Several of the many hormones mentioned above have the potential to serve as useful clinical biomarkers, however much research is still needed. Strong attention to the evolutionary perspectives and to the stage of the disease should be kept in this research. In every endocrinologically study of AN, it is not enough to include BMI in the interpretation but also the current nutritional status, e.g. expressed as short term relative weight change over the past 1–4 weeks and levels of exercise needs to be considered. Potential biomarkers should also be related to the plasma concentration of classical metabolic parameters such as T3 and insulin. Several ‘new’ peptides remain to be studied in AN, e.g. kisspeptin, gonadatropin-inhibitory peptide, and amide-related peptide-3, myostain, follistatin and PAPP-A. The important animal experimental data on oxytocin remains to be translated to human studies. We need biomarkers firstly to explore metabolism in AN to open new avenues of therapeutic targets, and secondly for predicting outcome and to ensure safe refeeding. A breakthrough in our understanding and treatment of this whimsical disease is still awaited.

### Limitations

This is a narrative review based exclusively on English language articles indexed in MedLine. Original studies and reviews from the past 15 years were mainly selected, but commonly referenced older publications were not excluded. The conclusions are influenced by the authors own experiences from more than 25 years of clinical work with AN, and are not generated by meta-analyses of systematic reviews. This weakens the evidence and should be taken into consideration.

## Declaration of interest

The author declare that there is no conflict of interest that could be perceived as prejudicing the impartiality of this review.

## Funding

This research did not receive any specific grant from any funding agency in the public, commercial or not-for-profit sector.
